# Potential Use of Castor Oil-Based Polyurethane Matrix Composite with Miriti Fiber Filling as Thermal Insulation Applied to Metal Tiles

**DOI:** 10.3390/polym17070892

**Published:** 2025-03-26

**Authors:** Waldemiro José Assis Gomes Negreiros, Jean da Silva Rodrigues, Maurício Maia Ribeiro, Douglas Santos Silva, Raí Felipe Pereira Junio, Sergio Neves Monteiro, Alessandro de Castro Corrêa

**Affiliations:** 1 Materials Engineering Program, Federal Institute of Education, Science and Technology of Pará—IFPA, Avenida Almirante Barroso, 1155, Marco, Belém CEP 66093-020, PA, Brazil; waldemiro.negreiros@ifpa.edu.br (W.J.A.G.N.); jean.rodrigues@ifpa.edu.br (J.d.S.R.); alessandro.correa@ifpa.edu.br (A.d.C.C.); 2 Federal Institute of Education, Science and Technology of Pará—IFPA, Estrada do Icuí Guajará, Ananindeua CEP 67125-000, PA, Brazil; mauricio.maia@ifpa.edu.br; 3 Military Institute of Engineering—IME, Department of Materials Science, Praça General Tibúrcio, 80, Praia Vermelha, Urca, Rio de Janeiro CEP 22290-270, RJ, Brazil; raivsjfelipe@ime.eb.br (R.F.P.J.); sergio.neves@ime.eb.br (S.N.M.)

**Keywords:** thermal performance, natural fibers, *Mauritia flexuosa*, thermal blanket, Amazon region

## Abstract

The Amazon biome’s climate, with annual temperatures above 30 °C and humidity over 90%, poses challenges for building thermally comfortable structures without expensive cooling systems. This study developed a castor oil-based polyurethane (PU) composite with miriti fiber (*Mauritia flexuosa*) as a roof thermal blanket, comparing its performance to fiber cement, ceramic, and metal tiles. Measurements were conducted over 136 days at the Federal Institute of Education, Science and Technology of Pará, Campus Belém. From August to October 2022, the fiber cement tile (CT) showed average thermal reductions of 5.9475 °C, 6.13388 °C, and 6.37368 °C, while the FCT coating had more modest reductions of 3.6634 °C, 3.63291 °C, and 3.60598 °C. In November and December 2023, the PU/miriti coating reached the highest reductions, 18.64058 °C and 17.88021 °C. Meanwhile, FCT recorded lower values of 1.74124 °C and 1.74721 °C. Observations show fiber cement allowed the highest heat transfer, whereas a metal tile combined with the PU/miriti composite provided better thermal performance than fiber cement and ceramic, meeting standards approval. The findings highlight the PU/miriti composite’s viability for roofing in hot, humid climates where maintaining lower indoor temperatures is essential. By reducing reliance on mechanical cooling, this technology can foster sustainable, cost-effective building practices in the region.

## 1. Introduction

The Brazilian Amazon region has a predominantly sunny climate, high temperatures, high rainfall, air instability, and high humidity [1], with an annual average temperature of 26.5 °C [2], which combined with heat islands resulting from urban occupation and climate change, contributes to an increasing risk of heat stress faced by local residents [3,4]. In particular, one should bear in mind that, in single-story buildings, heat is transferred from the external environment mainly through the roof (70%) [5].

Belém, the largest metropolis in the Eastern Amazon, located in the northeast of the state of Pará and 160 km from the equator [6], exhibits the poorest thermal performance of Brazilian buildings [7,8]. This is because it has a mostly perpendicular incidence of solar radiation directly on the roof of buildings [9], worsened by heat absorption by conventional construction materials [10], which directly influences local thermal comfort [11]. Moreover, factors that affect the thermal performance of buildings vary according to the type of roof used [12]. The energy performance of a building is closely linked to its thermal performance, i.e., a high thermal load generates greater thermal discomfort, which contributes to an increase in energy consumption for refrigeration [13]. Studies on the thermal and energy performance of buildings currently include the use of eco-friendly materials [14,15], such as natural fiber composites. These studies represent an emerging field of research in materials science and technology [16] and are gradually being developed due to the growing global energy crisis and ecological sustainability [17].

Natural lignocellulosic fibers (NLFs) have several advantageous characteristics that make them promising candidates compared to synthetic fibers in the manufacture of composites, such as abundance, low density, affordable cost, processing flexibility, low abrasion, low toxicity, biodegradability, high shock absorption and adaptable properties [18]. Several researchers have addressed the use of composite materials with potentially applicable NLFs in engineering. Freires et al. [19] investigated the mechanical properties of polyester matrix composites reinforced with crocheted miriti (*Mauritia flexuosa*) fiber fabrics. Different fabric weights, fiber alkali treatments, and polyester matrix viscosities were analyzed, with some composites undergoing post-curing at 60 °C. The specimens were fabricated using vacuum-assisted manual rolling following a 2^4^ factorial design and tested according to ASTM D3039. The results indicated that fiber alkali treatment and matrix viscosity significantly influenced the tensile strength and elastic modulus. The highest tensile strength (13.02 MPa) was observed in composites with alkali-treated fibers, fabric weight of 473.9 g/cm^2^, high-viscosity matrix, and post-curing. The best modulus of elasticity (1.59 GPa) was obtained in samples without fiber treatment, lower-weight fabric, high-viscosity matrix, and post-curing.

Senthilkumar et al. [20] investigated hybrid composites reinforced with olive leaf fiber (OTL) and pineapple leaf fiber (PALF) at different weight ratios (50:50, 30:70, and 70:30). The results showed that epoxy composites reinforced with pure PALF exhibited the highest flexural strength (44.39 MPa) and modulus (4.82 GPa). Hybridization with OTL reduced flexural properties, with strength ranging from 16.41 MPa to 28.95 MPa and modulus between 2.04 GPa and 3.22 GPa. However, the 3OTL/7PALF hybrid composite demonstrated an 8.4% higher flexural modulus than pure OTL composites. The impact strength was 3 to 8 times higher in hybrid composites compared to pure OTL-reinforced epoxy, highlighting the benefits of PALF addition.

Bollino et al. [21] investigated the effect of chemical treatments on natural fiber-reinforced composites (NFRCs) made with hemp fibers treated with NaOH or (3-Glycidyloxypropyl) trimethoxysilane (GPTMS). The goal was to reduce moisture adsorption and improve mechanical performance. Moisture adsorption and tensile tests were conducted to evaluate the impact of chemical treatment, reagent concentration, and temperature. The 5 wt% treatments of both NaOH and GPTMS were the most effective, reducing water uptake from 7.74% to 6.46% (NaOH) and 5.58% (GPTMS) at room temperature and from 9.67% to 8.19% (NaOH) and 8.13% (GPTMS) at 50 °C. Mechanical tests before and after moisture exposure showed that water uptake primarily reduced stiffness by 50% (NaOH) and 60% (GPTMS), while the load-bearing capacity remained largely unaffected. Among the treatments, 5 wt% GPTMS proved to be the most effective in preventing failure caused by water absorption.

Souza et al. [22] evaluated epoxy matrix composites reinforced with 10, 20, and 30 vol% of continuous and aligned caranan fibers through tensile tests, TGA, and DSC. Composites with more than 10 vol% fibers showed significant improvements in elastic modulus, toughness, and strength compared to neat epoxy. The 30 vol% composite was 50% stiffer, 130% tougher, and 100% stronger, confirming its effectiveness as reinforcement. The TGA analysis indicated the highest onset degradation temperature (298 °C) and the lowest mass loss (36.8%) for the 30 vol% composite, which also exhibited the highest degradation peak (334 °C). The glass transition temperature (T_g_) ranged from 63 °C (DSC) to 113 °C (DMA).

Junio et al. [23] evaluated the influence of carnauba fibers (*Copernicia prunifera*) morphology on composite properties. Optical microscopy (OM) was used to assess fiber cross-section and diameter variation, enabling density measurement (1.13 ± 0.22 g/cm^3^) via the Archimedes method. Tensile tests and Weibull analysis were conducted to determine mechanical properties, revealing an average tensile strength of 64.7 ± 40.9 MPa, Young’s modulus of 1.37 ± 0.80 GPa, and elongation of 5.59 ± 1.60%. SEM analysis exposed surface and cross-sectional defects, which may influence mechanical performance.

Silva et al. [24] evaluated the thermochemical and structural properties of raffia fiber fabric using TGA, DSC, FTIR, XRD, lignocellulosic analysis, and SEM. TGA revealed moisture loss at 52 °C and thermal degradation starting at 245 °C. DSC detected an endothermic peak at 56 °C and two exothermic peaks at 298 and 336 °C associated with fiber constituents. FTIR confirmed the presence of cellulose, hemicellulose, and lignin, while XRD showed a crystallinity index (CI) of 66.04% and microfibrillar angle (MFA) of 7.24°. The raffia fabric had a moisture content of 9.47% and a density of 0.95 g/cm^3^, with a chemical composition of 52% cellulose, 25% lignin, and 14% hemicellulose. SEM analysis revealed porous surface characteristics. The study concludes that raffia fiber fabric demonstrates properties comparable to other natural fiber composites, making it a promising material for engineering applications and polymer reinforcement.

Paiva et al. [25] proposed an alternative expedient experimental setup to evaluate the thermal insulation performance of corncob particle boards. According to the authors, testing the thermal insulation performance in situ under real thermal and hygrometric conditions, using more realistic sample dimensions, simultaneously testing several samples, and continuously monitoring the thermal behavior of a product for several days are some advantages of this proposed technique. It has proven to be accurate and versatile.

Charca et al. [26] evaluated the thermal properties of natural Ichu fibers characterized according to ASTM C177. The results showed that the thermal conductivity ranges from 0.047 to 0.113 W/m·K for carpets with unidirectional oriented fibers, with fine Ichu having the lowest values. According to the authors, in order for fine Ichu fiber to be cost-competitive per unit of thermal resistance, the density of the fiber mat was reduced by arranging the fibers randomly; the results showed a significant reduction in density without significantly increasing thermal conductivity.

Wei et al. [27] investigated the effects of high-frequency heating, board density, particle size, and ambient temperature on the properties of RSTIB. Optimal physical and mechanical properties were achieved with 14% particle moisture content, 250 kg/m^3^ board density, and L-type particle size. The thermal conductivity of the insulation boards was low, ranging from 0.051 to 0.053 W/(m·K). High-frequency hot pressing significantly reduced pressing duration compared to conventional methods and resulted in higher internal bonding strength (IB).

Verma et al. [28] evaluated the effect of Passive Methods (PMs) (e.g., window layers, colors, infiltration, insulation, and orientation) on controlling heat gain in buildings in 18 Indian cities with different climates. Three scenarios were analyzed: PMs alone, PMs with a reflective roof (RR), and PMs with a reflective roof and wall (RRW). The study also evaluated the impact of reflective surfaces on insulation thickness requirements. Using the Non-Dominated Genetic Algorithm (NSGA-2), the findings reveal a significant reduction in energy demand, with HVAC energy savings of 6–29% annually. A coupled system of PMs and RR further increased energy savings to 11–32%, varying by climate, while reducing insulation thickness requirements. The optimal window solution across all climates was a 6 mm/13 mm double-layer green air window.

The species *Mauritia flexuosa* L. (*Arecaceae*), popularly known as buriti, is one of the most prevalent palm trees in the Amazon region [29] and demonstrates great potential for the production of high-quality fibers for reinforcement composites [30,31,32]. Miriti has been extensively used as a source of food and beverages, as well as a component of cosmetics and artisanal items [33,34]. However, it is the use of miriti fiber as a raw material for artisanal productions that most effectively combines the livelihood of traditional communities with the sustainable exploitation of this non-timber forest resource [35].

The association of an NFL, like the miriti, with a renewable matrix is a desirable combination for the fabrication of sustainable bio composites. Vegetable oils, in addition to being derived from a renewable source, have significant importance in the production of biopolymer composites [36]. Among vegetable oils, castor oil stands out as an excellent alternative due to its low toxicity, biodegradability, cost-effectiveness, suitability for industrial application, and availability [37].

Furthermore, studies proposed castor oil as a source of polyol to replace petroleum derivatives [38]. Castor oil is extracted from the *Ricinus communis* plant, native to tropical Asia and Africa [39], and abundantly found in several regions with a tropical climate, such as in the Brazilian territory, which drives its increasing growth and use [40,41]. The adoption of castor oil as a natural polyol appears to be a promising choice to promote sustainability and reduce dependence on non-renewable resources in the industry. It is worth mentioning that Brazil is the third largest producer of castor oil in the world [42].

Polyurethane (PU) is currently widely used as a matrix in composite materials reinforced with NLFs due to its greater chemical compatibility [43]. PU can be combined to generate unique physical and chemical properties [40]. The thermal conductivity of PU composites reinforced with NLFs varies between 0.03 and 0.1 W/m·K [44], as low as that of the main industrial thermal insulators [45], which guarantees good thermal performance for mechanical and thermal insulation [46,47,48].

In this context, this work aims to develop a composite from two-component PU based on castor vegetable oil reinforced with miriti fiber to be used as a thermal blanket and evaluate the differences between external and internal temperatures with different covers.

## 2. Materials and Methods

### 2.1. Materials

**Figure 1:**
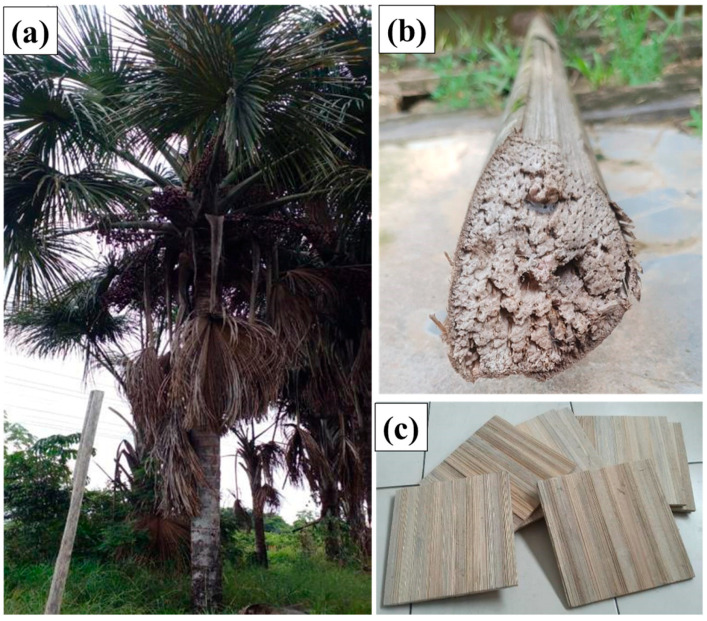
(**a**) Miriti plant; (**b**) miriti petioles, as sold; and (**c**) cut miriti.

The polymeric matrix used was the two-component vegetable PU adhesive resin, originating from castor oil (*Ricinus communis*), supplied by the company IMPERVEG^®^ (Aguaí, Brazil) with the specification of AGT 1315, supplied in two components called compound A (pre-polymer) and B (polyol) in the proportion of 1 part of A to 1.5 parts of B. Miriti (*Mauritia flexuosa*) fibers were purchased at the local market in the city of Abaetetuba, state of Pará, Brazil. Figure 1 (a) illustrates the miriti plant, (b) illustrates the miriti petioles, as sold, and (c) illustrates the cut miriti.

**Figure 2:**
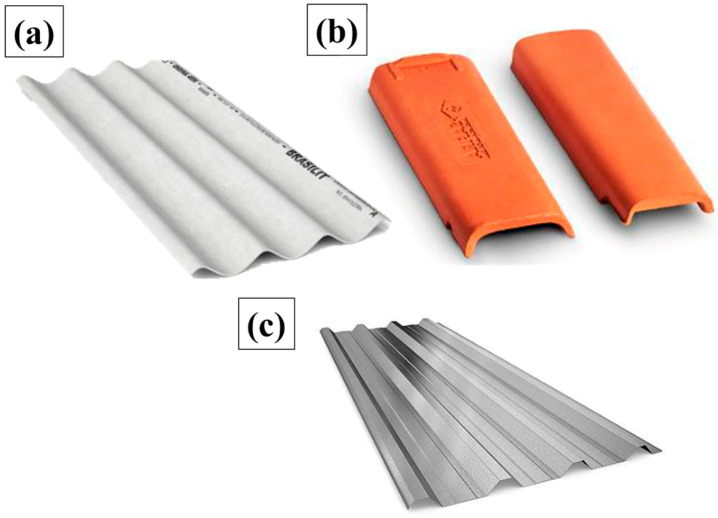
(**a**) Fiber cement tile (BRASILIT, 2024); (**b**) ceramic tile (CERAMICASAFIRA, 2024); and (**c**) galvanized tile (REDEOCA, 2024).

Three types of tiles were used in this research. Fiber cement tiles (FCTs) have characteristics, according to Table B.3 NBR 15575 [49], comprising dimensions of 220 cm long by 120 cm wide and 0.5 cm thick, a density of 1800 kg/m^3^, a thermal conductivity between 0.65 and 0.95 W/m·K, and a specific heat of 0.84 kj/kg K. Ceramic tile (CT) has characteristics, according to Table B.3, comprising dimensions of 40.1 cm long, 23.3 cm wide and 1.0 cm thick, as well as an average yield of 16.5 T/m^2^, a density of 1500 kg/m^3^, a thermal conductivity between 0.70 and 1.05 W/m.k, and a specific heat of 0.92 kj/kg K. Trapezoidal-shaped galvanized metal tile (TG) has the following dimensions: 180 cm long, 100 cm wide, and 8.4 cm thick. The thermal blanket was produced to cover the lower face of the metal tile, as shown in Figure 2.

### 2.2. Methods

#### 2.2.1. Manufacturing Process of the Composite Applied to Galvanized Tiles

**Figure 3:**
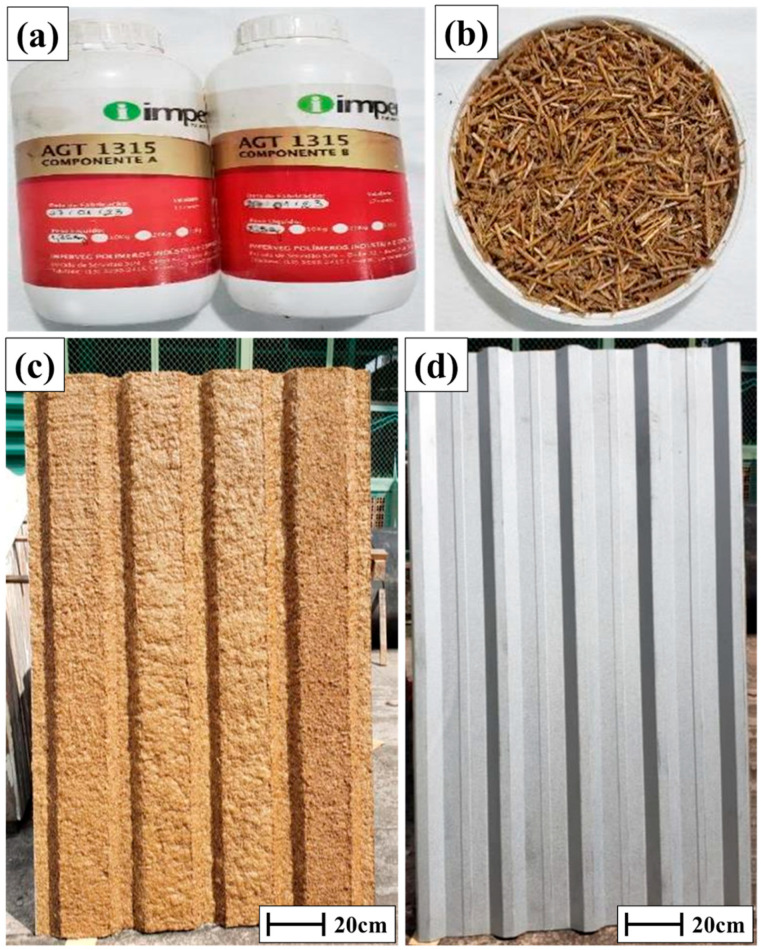
(**a**) Vegetable polyurethane; (**b**) miriti fiber; (**c**) metal tile, upper face; and (**d**) lower face.

**Figure 4:**
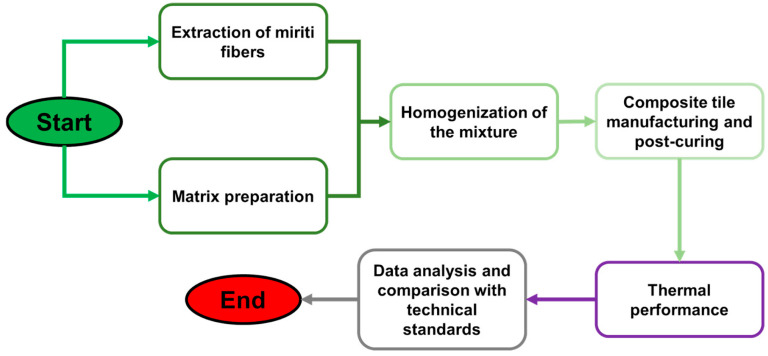
Flowchart of the experimental procedure followed in the present work.

In the preparation of the castor oil-based matrix, a mass fraction value was experimentally defined as 13.36% fiber and 86.63% resin, as well as 2623.78 g of compound A and 3935.67 g of compound B. The miriti fiber was crushed in a blender, sieved through a wooden sieve 70 cm, and heated in an electric oven at a temperature of 90 °C for 30 min to eliminate moisture. A total of 1011.60 g of miriti fiber was used to make the thermal blanket. Figure 3 shows the materials used. The complete flowchart of the experimental procedure is shown in Figure 4.

#### 2.2.2. Experimental Module

**Figure 5:**
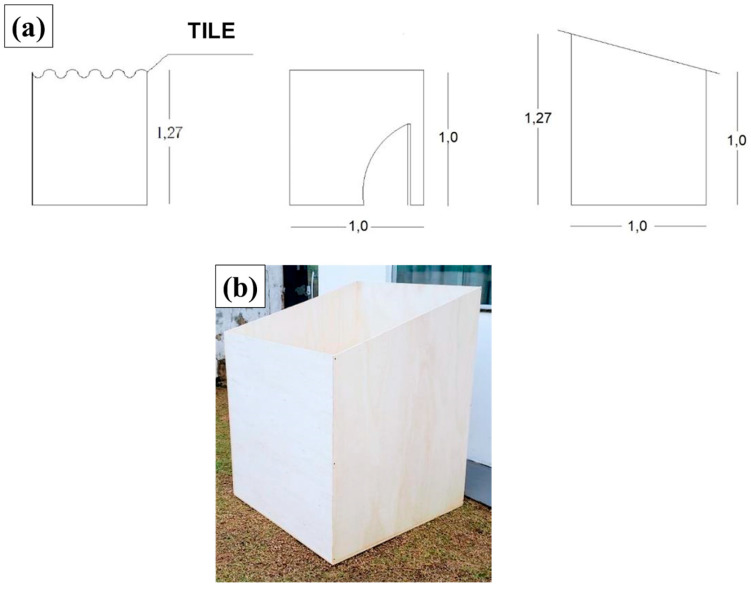
(**a**) Floor plan of the experimental module and (**b**) experimental module (without cover).

Figure 5a,b illustrate the experimental modules used in this research, with dimensions of 1 m wide, 1 m long, and 1 m and 27 cm high (1 m × 1 m × 1.27 m), arranged with a minimum distance of 1.5 m between each module.

Thermal performance evaluations were divided into two stages throughout the year. The first stage was carried out from 1 August to 31 October, in which the experimental modules were covered with FCT and CT. The second stage was from 1 November to 14 December, in which the experimental modules were covered with FCT and miriti polyurethane matrix (MPM).

For data acquisition and analysis, a logger based on open-source electronics (Arduino) was designed to monitor experimental modules and meet characteristic demands such as autonomy, reduced size, and low cost [50,51,52]. In the construction of the device, an Arduino board, Uno R3, a 400-hole protoboard, dht11 sensor, DS18B20 sensor, DS3231 module (RTC module—Real-Time Clock), SD card module, and 5 mm LDR 5528 photoresistor sensor were used. Communication between the device and the user was made through a liquid crystal display (LCD). This was necessary to monitor any variable instantly. The code written in the Arduino IDE, using the C/C++ language, was divided into two functions. The first function decodes the sensor data, while the second function is responsible for transferring the data collected from the sensors to a CSV file and storing it on the SD card. The update rate, that is, the time interval between each sensor observation, was set at 60.000 milliseconds (1 min). The collected data were later processed by the R/RStudio software.

To evaluate the thermal performance of the tiles tested, criteria for maximum temperature values are described in NBR 15575 [49], which considers these temperatures as the maximum daily air values inside the building in degrees Celsius.

#### 2.2.3. Statistical Analysis

A one-way analysis of variance (ANOVA) was performed to assess the statistical difference in the external and internal temperature across different types of tiles, followed by a post hoc Tukey test for pairwise comparisons under normality and homogeneity of variances, as verified by the Shapiro–Wilk and Levene tests. In instances where the data were normally distributed but variances were unequal, a one-way ANOVA with Welch’s correction was conducted, followed by the Games–Howell post hoc test.

## 3. Results and Discussion

### 3.1. Experimental Scalability

The adoption of a 1 m^3^ module does not directly reflect the typical size of a real building but constitutes a laboratory scale that facilitates the control and comparison of variables. In thermal comfort research and roofing material evaluation, it is common to use reduced modules to isolate the effect of the roof system and simplify data collection. This way, it is possible to monitor the variation in internal and external temperature without the interference of other factors that would be present in a large building, such as door and window gaps, thermal inertia of thick walls, or air conditioning equipment.

Furthermore, a smaller volume allows for repetitive and lower-cost testing, as it requires less material to build the module and less collection time to detect differences in performance. The cubic geometry and standardized size also help keep the roof area-to-volume ratio similar across different studies, making it easier to compare results. Although the 1 m^3^ module does not faithfully reproduce the conditions of a residence or building, it functions as a prototype to evaluate and compare, on a reduced scale, the capacity of each roofing system to attenuate heat.

Thus, the justification for scale lies in the practical feasibility of testing different materials under controlled conditions, isolating as many as possible variables that could mask the thermal performance of the roof. When the effectiveness of a composite (such as MPM) is proven in small modules, studies are then carried out on a larger scale or in field applications, validating whether the observed gains are maintained in real buildings.

### 3.2. Experimental Module Covered with FCT

**Figure 6:**
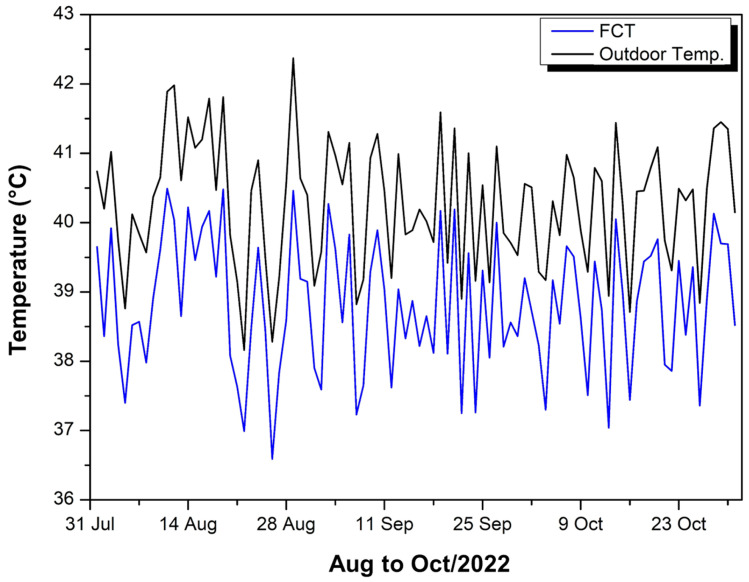
FCT internal temperature—Aug to Oct/22.

Figure 6 shows the curve of maximum temperatures inside the experimental module covered with FCT.

The blue curve represents the maximum temperatures recorded inside the experimental module covered with FCT, while the black curve shows the maximum external ambient temperatures. Observing the data from August to October 2022, it is noted that, in general, the internal temperature (FCT) remains below the external temperature but with relatively small differences, in the range of approximately 1 to 2 °C. The highest maximum temperature measured inside the module was 40.49 °C and the lowest, 36.59 °C, resulting in a variation of 3.9 °C over the period and an average of 38.81 °C.

Throughout August, the difference between the external and internal temperatures varied between 1.04 °C and 1.48 °C; in September, from 1.02 °C to 1.99 °C; and in October, from 1.02 °C to 1.94 °C. This variation suggests that the FCT manages to slightly reduce internal temperatures relative to external ones, but the thermal attenuation is not very pronounced, remaining close to 1–2 °C most of the time.

It is also possible to observe a certain daily fluctuation, in which peaks of external heat are accompanied by an increase in internal temperature, although there is always an interval (or “offset”) that indicates some degree of insulation provided by the FCT. The internal curve (blue) shows a smoother behavior, without oscillations as wide as the external curve (black), which may indicate a small thermal inertia capacity of the roofing material or the internal environment.

Overall, the analysis of the period shows that, even on the hottest days, the FCT keeps the inside of the module a few degrees below the external ambient temperature, with the internal maximum always lower than the external one. Still, the difference does not exceed 2 °C in most measurements, which means that, although there is an insulation effect, it is relatively limited.

**Figure 7:**
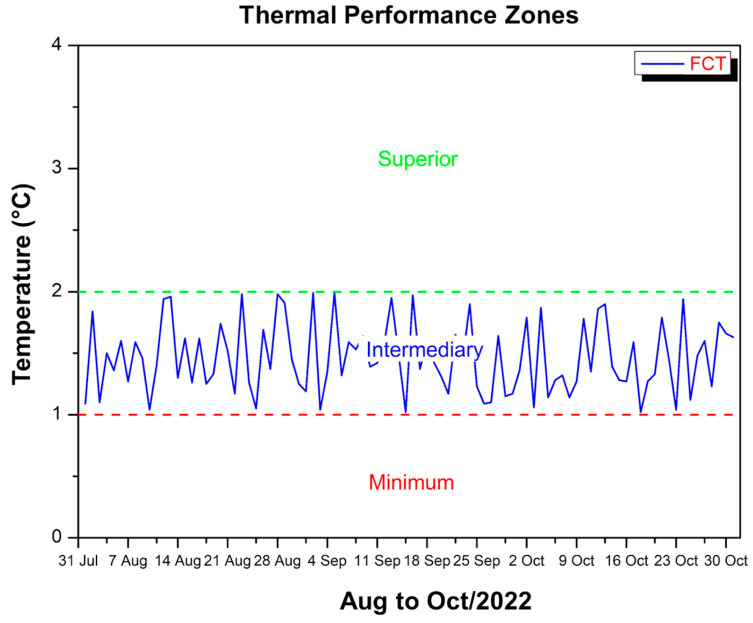
Thermal performance criterion FCT—Aug to Oct/22.

Figure 7 shows the difference between the maximum temperatures inside the FCT-coated module and the external temperature over the period from August to October 2022.

The vertical axis indicates the value of this difference (in °C), while the horizontal axis covers the time interval in days. The dotted lines in green (upper zone) and red (minimum zone) delimit thermal performance ranges so that whenever the blue curve is above the red line and below the green line, it means that the inside of the module remains cooler than the external environment in a specific range of 1 to 2 °C. It can be seen that the curve does not cross the zero line at any time, confirming that the internal temperature does not exceed the external temperature, in accordance with the standard that requires the internal temperature to always be lower than the external temperature. This thermal difference fluctuates slightly over the period but remains within the established ranges, suggesting that the FCT plays an effective attenuation role, keeping the interior of the module between approximately 1 and 2 °C below the ambient temperature on the days analyzed. This effect is due in part to the material’s ability to reflect some of the incident solar radiation and to reduce heat transfer by conduction. The composition and thickness of the coating, as well as the existence of possible air layers between the FCT and the main structure, help to delay the flow of heat into the interior.

Furthermore, the presence of fibers or additives in the coating can favor the dispersion of heat along the surface, preventing the heat from concentrating at specific points. If the FCT has low thermal conductivity or some reflective layer, it reduces the amount of radiation absorbed by the roof, which results in less heating of the internal environment. The fact that this difference of 1–2 °C was maintained during the observed period indicates that the material is relatively stable under everyday climatic conditions as long as there is no significant degradation.

However, some factors can affect long-term thermal performance. Moisture absorption, for example, can modify the thermal properties of the coating, making it more conductive or prone to adhesion failure. Prolonged exposure to ultraviolet (UV) radiation and temperature variations can degrade the organic components of the FCT, reducing its reflectivity or creating cracks that facilitate heat exchange. The accumulation of dirt or debris on the surface can also reduce the ability to reflect solar radiation, further heating the interior. Therefore, the durability of FCT depends both on its formulation characteristics and on periodic maintenance to preserve its thermal properties over time.

**Figure 8:**
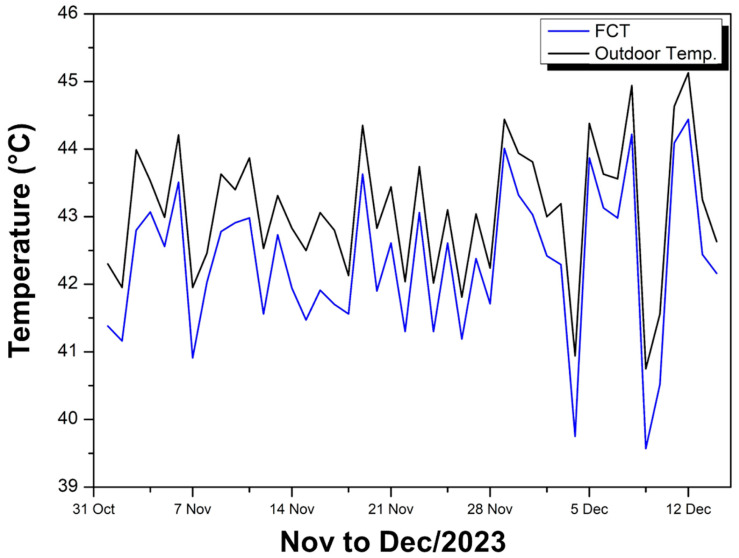
Ambient and internal temperature FCT—Nov to Dec/23.

Figure 8 shows the maximum temperatures recorded between late October and mid-December 2023, comparing the interior of the experimental module covered with FCT (blue curve) and the external environment (black curve).

The highest internal temperature value reached 44.44 °C, while the lowest value was 39.57 °C, resulting in a variation of almost 5 °C and an average of 41.41 °C in the period analyzed. When comparing this interval to external oscillations (black curve), it can be seen that the variation in the external environment is generally greater and reaches peak values slightly higher than those recorded inside the module. In other words, although the internal temperature follows the external fluctuations, it maintains a slightly smaller oscillation range, which indicates some degree of thermal attenuation provided by the FCT coating.

Several factors contribute to this internal variation of 4.87 °C. Firstly, direct and diffuse solar radiation raises the module temperature throughout the day, and the coating’s ability to reflect or absorb heat influences how quickly the interior heats up. Then, the thermal conductivity of the FCT, combined with the thickness and possible air layers between the coating and the structure, helps to slow the passage of heat inward but does not block it completely. Another relevant aspect is the thermal inertia of the module: materials with greater mass or heat storage capacity tend to heat up and cool down more slowly, resulting in less abrupt internal variations than external ones.

Additionally, possible air infiltrations or small gaps in the module can allow convective heat exchange, increasing or reducing the internal temperature depending on external conditions. Finally, moisture present both in the coating and inside the module can alter the rate of heat exchange, as water evaporation and condensation influence the amount of energy dissipated. Together, these factors explain why the 4.87 °C variation occurs inside the module while remaining lower than the external temperature range.

On most days, the external curve remained above the internal one, confirming that the FCT kept the interior of the module at temperatures slightly lower than the external environment. Even so, the difference between the curves varied from just 0.43 °C at the points where they almost overlapped to 1.19 °C at the interval of greatest discrepancy, presenting an average difference of 0.748 °C. This behavior indicates that, although the coating reduces the heat inside the module, the thermal attenuation remains at relatively low values, close to 1 °C. It is possible to note that, even on the hottest days, the internal temperature did not exceed the external temperature, which suggests that the FCT continues to play an insulating role, albeit of moderate intensity, keeping the interior of the module below the external environment, but without major fluctuations in difference over the period.

**Figure 9:**
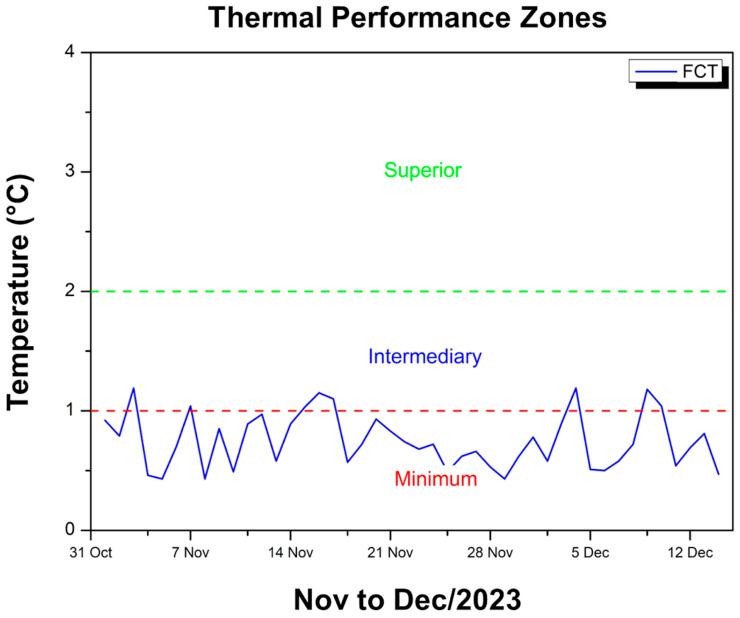
Thermal performance criterion FCT—Nov to Dec/23.

Figure 9 shows the difference between the maximum temperature inside the module, coated with FCT, and the external temperature.

It can be seen that the internal environment remained below room temperature throughout the period, as required by the standard. The blue curve oscillates between approximately 0.43 °C and 1.19 °C, remaining most of the time within the range considered minimum (up to 1 °C) by NBR 15575 [49], which indicates that the thermal insulation provided by the FCT reduces the internal temperature, but moderately. At times, the curve slightly exceeds 1 °C, falling into the intermediate category according to the criteria of the same standard, but these peaks are punctual. In general, thermal performance is mostly in the minimum zone, demonstrating that, although the FCT manages to keep the interior of the module at temperatures lower than the external environment, this difference is not very significant on most days. This suggests that the coating effectively acts as a thermal barrier, albeit with limited intensity, ensuring that the internal temperature remains, on average, less than 1 °C below the external temperature, with occasional elevations to the intermediate range at specific points.

### 3.3. Experimental Module Covered with CT

**Figure 10:**
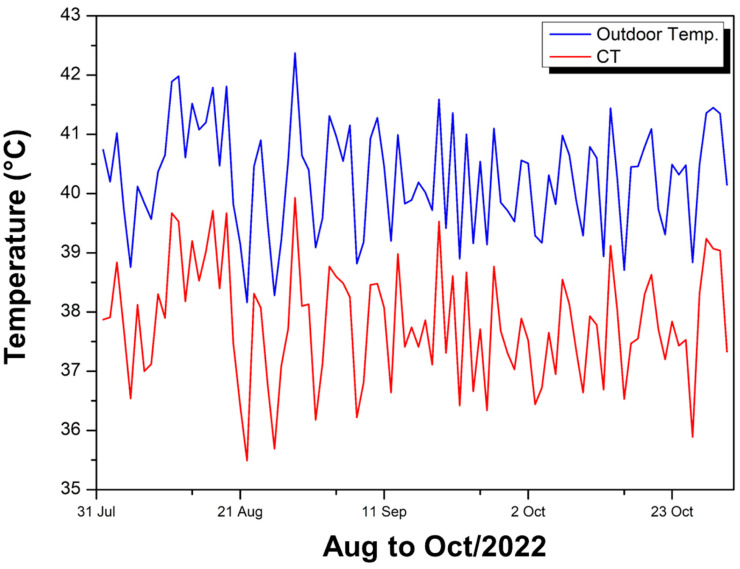
Internal temperature FCT—Aug to Oct/22.

Figure 10 compares the maximum external ambient temperatures (blue curve) with those inside the experimental module covered with CT (red curve) throughout August to October 2022.

It is observed that the internal temperature remains consistently lower than the external temperature, with a higher maximum value of 39.93 °C and a lower value of 35.49 °C, resulting in an average of 37.79 °C. In August, the difference between the external and internal temperatures varied between 2 °C and 2.87 °C; in September, between 2.01 °C and 2.91 °C; and in October, between 2.03 °C and 2.99 °C. These difference ranges, close to 2 to almost 3 °C, show that the CT coating provides a more pronounced thermal attenuation compared to other solutions with a smaller reduction range.

The red curve, in general, follows the behavior of the blue curve but shifts to lower values, indicating that, on hotter days, there is still a heat gain inside the module, although attenuated by the coating. Daily fluctuations show that when the external temperature peaks above 40 °C, the internal temperature remains in the range of 35–39 °C, with a reduction margin of approximately 2–3 °C. This behavior suggests that the CT provides effective thermal insulation, ensuring that, even in the hottest periods, the internal environment remains noticeably cooler than the external environment.

**Figure 11:**
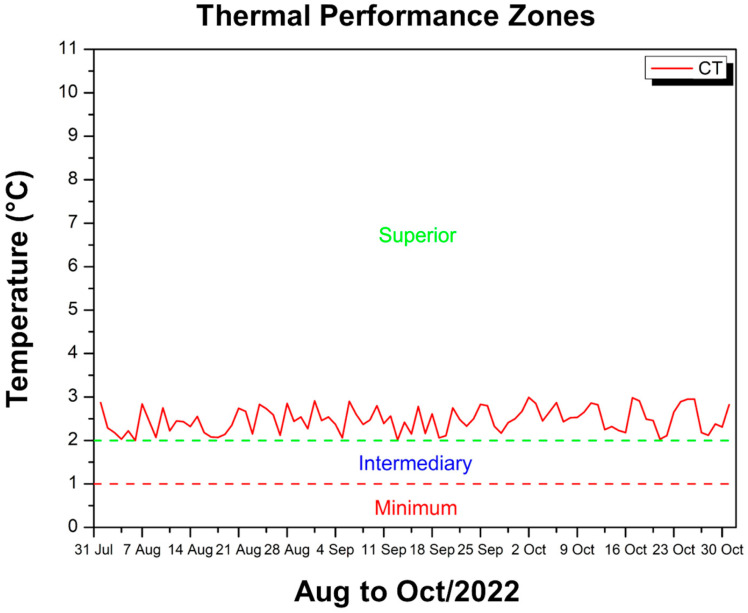
Thermal performance criterion CT—Aug to Oct/22.

Figure 11 shows the difference between the maximum temperature inside the experimental module, coated with CT, and the external ambient temperature, allowing us to verify whether the internal environment remained cooler than the external one.

The values on the vertical axis are between approximately 2 °C and 2.99 °C, therefore remaining within the range classified as “superior” according to the thermal performance criteria established by NBR 15575 [49]. This means that, over the period from August to October 2022, the CT coating ensured a reduction of at least 2 °C in relation to the external temperature on most days, reaching values close to 3 °C at times. The red line, which represents this difference, always remains above the minimum and intermediate zone, showing that the coating provides more effective thermal insulation, keeping the inside of the module considerably cooler compared to the external environment. Therefore, even on days when the external temperature reaches high levels, the use of the CT provides the module with sufficient heat attenuation to meet the requirement that the internal temperature remain below the external temperature, with a performance classified as superior by the standard.

### 3.4. Experimental Module Covered with MPM

**Figure 12:**
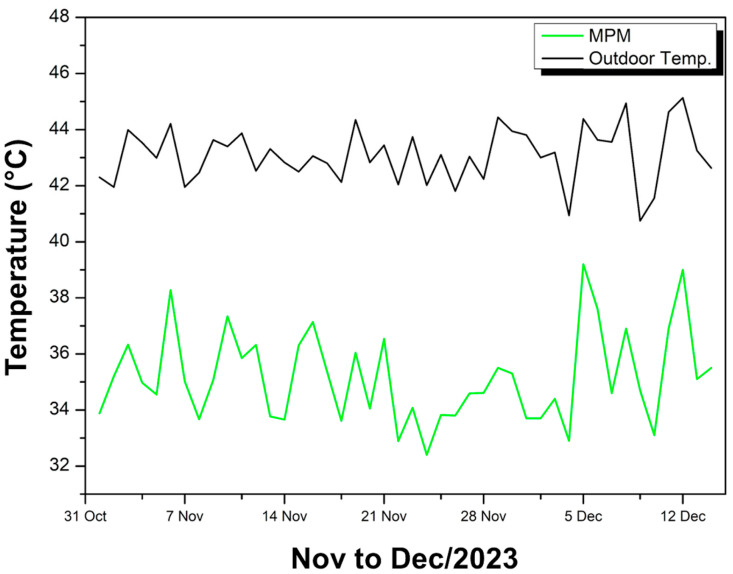
Ambient and internal temperature MPM—Nov to Dec/23.

Figure 12 compares the maximum external ambient temperatures (black curve) with those recorded inside the experimental module covered with MPM (green curve) over 44 days between late October and mid-December 2023.

It can be seen that the internal temperature remains consistently lower than the external temperature, with values ranging from 32.40 °C (lowest) to 39.20 °C (highest), resulting in an average of 35.16 °C inside the module. Meanwhile, the external temperature exceeds 45 °C at times, maintaining a very marked difference in relation to the internal temperature.

The discrepancy between the two curves ranges from 5.18 °C to 10.11 °C, with an average difference of 9.92 °C, which indicates significant thermal insulation provided by the MPM. In other words, the coating ensures that, even on very hot days, the interior of the module remains substantially cooler than the external environment, reaching up to 10 °C below the external temperature at certain peaks.

The green curve (MPM) displays daily oscillations that follow, on a smaller scale, the peaks and valleys of the black curve (external temperature), but always shifted toward lower values. This behavior reinforces the efficiency of the MPM in attenuating heat transfer to the interior, resulting in a more stable internal environment that is less subject to the sudden temperature increases observed outside. Thus, the MPM coating is highly effective in reducing heat gain in the experimental module, ensuring superior thermal performance throughout the entire evaluated period.

**Figure 13:**
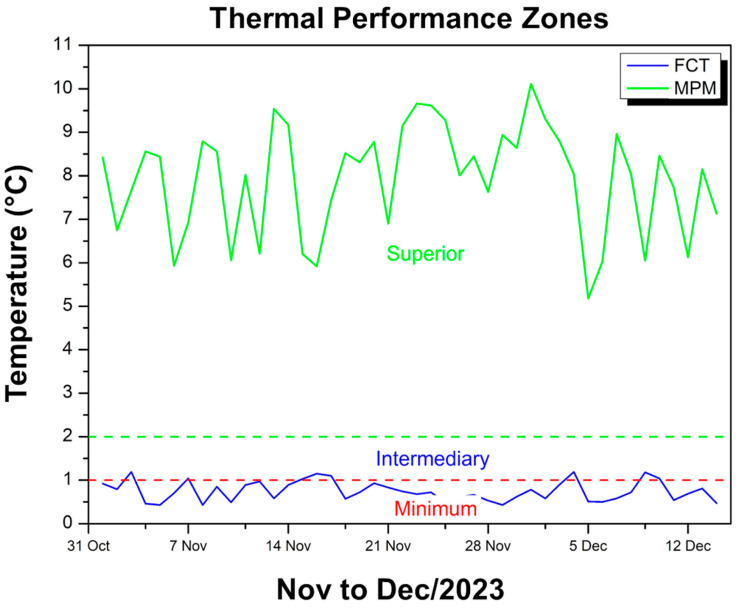
MPM thermal performance criteria—Nov to Dec/23.

Figure 13 shows two temperature difference curves in relation to the external environment: one in blue, referring to the FCT coating, and another in green, referring to the MPM coating.

It can be seen that the blue curve (FCT) oscillates predominantly in the range of 0.43 °C to approximately 1.2 °C, which, according to the NBR 15575 classification [49], falls within minimum thermal performance or, at some points, intermediate. In contrast, the green curve (MPM) remains at much higher values, ranging from approximately 5.18 °C to 10.11 °C, thus classifying it as superior performance according to the same normative criteria. This disparity shows that while the FCT attenuates the internal temperature in relation to the environment, albeit in a limited way (close to 1 °C), the MPM provides a much more significant reduction, reaching a difference of up to 10 °C. Thus, throughout the period evaluated (November to December 2023), the MPM coating stands out for keeping the maximum temperature inside the module considerably below the external temperature, with an attenuation that the standard classifies as superior, while the FCT presents a more modest effect, even though it meets the requirement of keeping the internal temperature below the external temperature.

### 3.5. Comparison of Internal Temperatures

#### 3.5.1. Comparison of Internal Temperatures of FCT and CT

**Figure 14:**
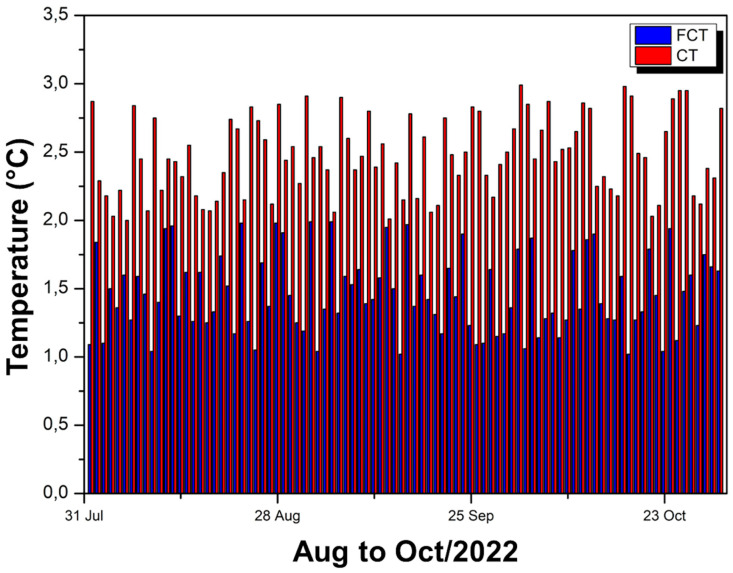
Comparison of internal temperatures of FCT and CT—Aug to Oct/22.

Figure 14 presents comparative bars that indicate the percentage reduction in internal temperature in relation to external temperature for two experimental modules: one covered with FCT (blue bars) and the other with CT (red bars) during the period from August to October 2022.

It is generally observed that the bars referring to CT (red) are higher than those of FCT (blue), revealing greater thermal attenuation capacity. On average, in August, the FCT module obtained a 3.66% reduction, while the CT module reached 5.95%; in September, the FCT reached 3.63% and the CT reached 6.15%; and in October, the FCT had 3.61% against 6.37% of the CT. These values show that CT reduces the internal temperature by approximately 2 to 3 percentage points more than FCT, resulting, in month-to-month comparisons, in approximately 1.6 to 2% better performance for CT when evaluating the specific difference between the two materials.

Over the three months, it can be seen that the FCT maintains an average reduction of around 3.6%, while the CT remains in the range of 6%, reinforcing the idea that the CT provides a more effective thermal barrier. Even with daily fluctuations (reflected in the variations of the bars), the trend remains consistent: the CT reduction curve remains higher, indicating that the interior of the module covered with this coating reaches lower temperatures compared to the module with FCT. Therefore, although both materials fulfill the role of reducing the internal temperature in relation to the external temperature, CT stands out for achieving a higher level of thermal performance, as demonstrated by the percentage differences from month to month.

**Figure 15:**
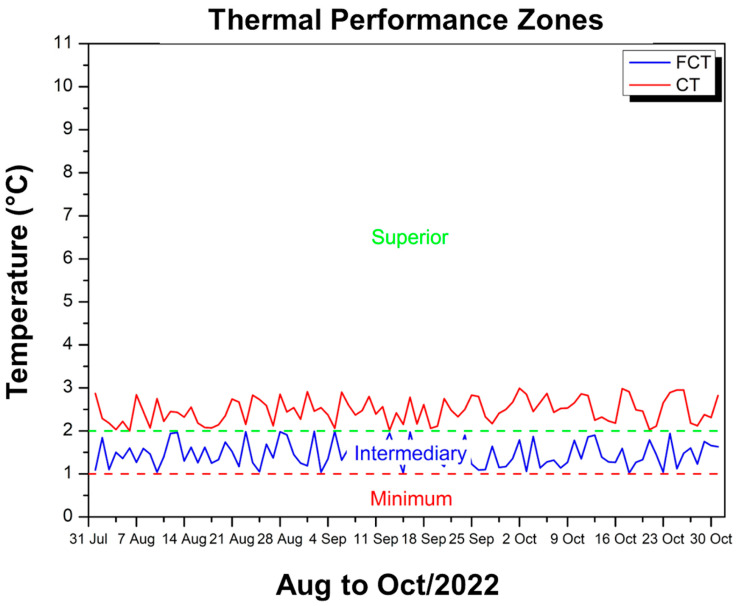
FCT and CT thermal performance criteria—Aug to Oct/22.

Figure 15 shows two temperature difference curves in relation to the external environment: one in blue (FCT) and the other in red (CT).

It is observed that, from August to October 2022, the FCT reduced the internal temperature by values between 1.02 °C and 1.99 °C, while the CT achieved differences of 2 °C to 2.99 °C, which means a more pronounced thermal attenuation. According to the criteria of NBR 15575 [49], the FCT is predominantly in the minimum to intermediate performance range, as it does not exceed 2 °C of reduction, while the CT remains in the upper range, as its differences reach almost 3 °C on certain days. Thus, although both meet the regulatory requirement of keeping the internal temperature below the external temperature, the CT demonstrates greater efficiency, with thermal performance 2.61% higher than the FCT in the period evaluated, evidenced by the higher position of the red curve in relation to the blue one along the graph.

#### 3.5.2. Comparison Between FCT and MPM Tile Coverage

**Figure 16:**
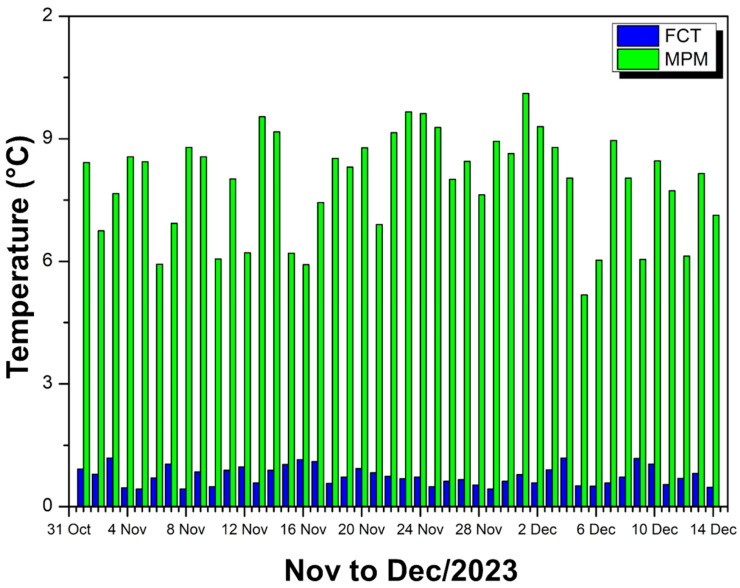
FCT and MPM thermal reduction—Nov to Dec/23.

Figure 16 shows comparative bars of percentage reduction in internal temperature in relation to external temperature for two experimental modules: one coated with FCT (blue bars) and the other with MPM (green bars).

It can be observed that, in the period from November to December 2023, the bars referring to MPM are consistently higher than those of FCT, indicating greater efficiency in thermal attenuation. On average, the module with FCT obtained only a 1.74% reduction, while the module with MPM achieved 18.39%, resulting in a difference of 16.94% in favor of MPM.

This significantly superior performance of MPM is evident in the daily comparison, as the green bars remain well above the blue bars throughout the period, showing that the MPM coating is able to reduce the internal temperature much more significantly. FCT, on the other hand, although it shows some attenuation, remains at relatively low values (in the range of 1 to 2%), demonstrating less effectiveness as a thermal insulator. Thus, the data confirm that the MPM provides a much more effective heat barrier, keeping the inside of the module considerably cooler compared to the outside environment.

**Figure 17:**
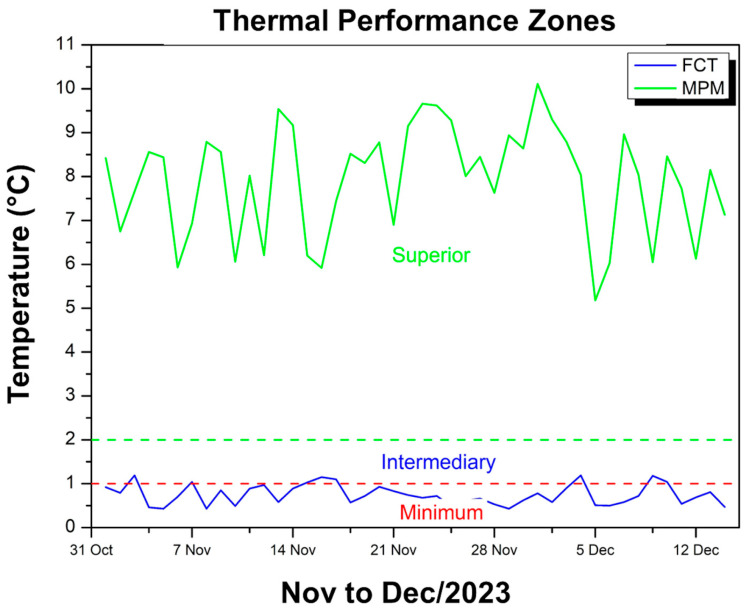
FCT and MPM thermal performance criteria—Nov to Dec/23.

Figure 17 shows two curves that indicate the difference between the maximum temperature inside the modules and the external temperature, one of which refers to the FCT coating (blue line) and the other to the MPM coating (green line).

It can be observed that, for the module with FCT, this difference remains in the range of 0.43 °C to 1.19 °C, which, according to the thermal performance criteria of the standard, corresponds predominantly to the minimum and eventually intermediate ranges. In contrast, the module with MPM exhibits much more expressive differences, ranging from 5.18 °C to 10.11 °C, thus classifying itself in the superior performance range. This means that while the FCT only provides a slight reduction in internal temperature compared to the external temperature, the MPM ensures a much more pronounced attenuation, keeping the inside of the module 5 °C to up to 10 °C cooler than the external environment. As a result, both meet the requirement of keeping the internal temperature below the external temperature, but the MPM demonstrates much more effective thermal insulation over the observed period, standing out as the best-performing option.

**Figure 18:**
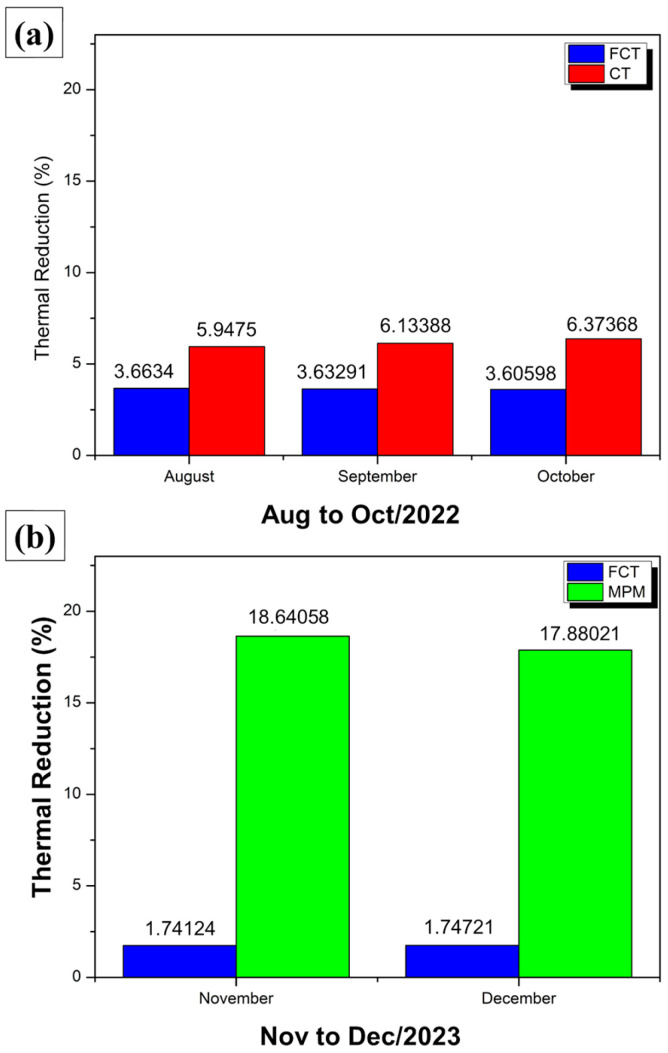
Thermal reductions observed in modules covered with CT and FCT.

Figure 18 presents two sets of data, divided into (a) and (b), which compare the thermal reduction obtained in experimental modules covered with different materials.

Figure 18a evaluates the months of August, September, and October, comparing the performance of CT (red bars) and FCT (blue bars). It can be seen that, in all months, CT achieves higher thermal reduction values, in the range of 5.95% to 6.37%, while FCT remains close to 3.6%. This indicates that, for this period, CT provides thermal attenuation approximately 2 to 3 percentage points higher than FCT, demonstrating greater effectiveness in reducing the internal temperature compared to the external temperature.

In Figure 18b, referring to the months of November and December, FCT (blue bars) and MPM (green bars) are compared. In this case, the difference in performance is even more pronounced: the FCT records only 1.74% and 1.75% thermal reduction, while the MPM reaches values of 18.64% in November and 17.88% in December. In other words, the MPM reduces the internal temperature in relation to the external temperature by around 18%, compared to less than 2% in the case of the FCT, highlighting a very large disparity between the two solutions.

Overall, the results indicate that, during the period evaluated, the CT outperforms the FCT in thermal insulation efficiency, keeping the inside of the module 2 to 3 percentage points cooler. On the other hand, the MPM presents an even more significant advantage compared to the FCT, with reductions of around 17 to 18 percentage points more. These findings reinforce that the choice of roofing material can have a significant impact on the thermal comfort of environments, with MPM demonstrating the best performance among the materials evaluated, followed by CT and, finally, FCT.

### 3.6. Analysis of Results

Table 1 consolidates the data obtained from both the temperature difference ranges and the average thermal reduction for each month and coating, indicating the average reduction value and the temperature difference range, in addition to the performance classification according to the defined ranges.

Analysis of the results indicates that in the months of August to October 2022, the FCT presented an average thermal reduction between 3.60 and 3.66 °C, while the temperature difference ranges showed values from 1.02 to 1.99 °C. The divergence between “range” and “average” is due to different calculation methods or the way the data are represented (peaks versus averages), but the FCT performance remains classified as Minimum/Intermediate, keeping the interior about 1 to 2 °C cooler than the exterior.

In November and December 2023, the FCT records lower averages (around 1.74 °C), with ranges between 0.43 and 1.19 °C, which confirms a lower performance compared to the previous period but still within the Minimum/Intermediate ranges.

Over the same period (August to October 2022), CT averages 5.95 to 6.37 °C, well above the 2.00 to 2.99 °C ranges observed in another dataset. This “Superior” rating suggests that the coating more effectively reduced the internal temperature, maintaining about 2 °C (or more) difference from the external environment.

The MPM, assessed only in November and December 2023, achieved averages of 17.88 to 18.64 °C of reduction, which is consistent with the range of 5.18 to 10.11 °C of difference in another type of measurement. This much higher value indicates that the MPM provided the greatest heat attenuation among the systems analyzed, falling within the “Superior” range.

In general, the FCT maintains intermediate performance, reducing the internal temperature by 1 °C to 2 °C, while the CT presents a higher gain (2 °C to 3 °C), and the MPM stands out for the greatest reduction of all (5 °C to 10 °C). These numerical discrepancies reflect the different ways of collection and representation (peaks, averages, ranges), but the relative positioning of the covers remains clear: the MPM offers the best insulation, followed by the CT, and the FCT obtains a smaller but still significant reduction compared to the external temperature.

Table 2 presents a comparison of the internal temperatures of different roofing materials and their thermal performances.

Reis et al. [53] analyzed metal tiles with EPS (expanded polystyrene) and PU insulation, considering heat transfer. PU showed better thermal performance than EPS at high temperatures, suggesting that this material may be a more efficient choice for thermal insulation in hot climates. Sampaio et al. [54] examined the internal and external temperatures of ceramic, fiber cement, and metal tiles without additional insulation. The results showed that the metal tiles reached surface temperatures of 53 °C, which confirms their worse thermal performance compared to the other materials analyzed.

Almeida et al. [55] compared metal and fiber cement tiles, highlighting that fiber cement tiles presented better thermal performance compared to metal tiles. This finding reinforces that the roofing material has a direct impact on the regulation of internal temperature. Bintarto et al. [56] evaluated metal tiles without coating, with epoxy coating, and with a layer composed of natural andesite powder and epoxy. The study concluded that the epoxy coating was effective in reducing the internal temperature, demonstrating that the type of coating can significantly improve the thermal insulation of the tiles.

Overall, Table 2 highlights that the use of thermal coatings and blankets can significantly improve the thermal performance of roofs, with the MPM presented in this study being the most efficient solution for reducing internal temperatures. In addition, materials such as fiber cement tend to offer better thermal insulation than metal and additional layers of coating can optimize the thermal performance of metal tiles.

### 3.7. Statistical Analysis

The Shapiro–Wilk tests indicated that the data satisfied the normality assumption required for ANOVA for external temperature (W = 0.987, *p* = 0.489), internal temperature of the FCT Tile (W = 0.975, *p* = 0.077), and internal temperature of the CT Tile (W = 0.992, *p* = 0.872). Furthermore, Levene’s test confirmed that variances were homogeneous, F(2, 273) = 0.426, *p* = 0.653.

The one-way ANOVA revealed significant differences in temperature among the groups, F(2, 273) = 158.601, *p* < 0.001. Post hoc pairwise comparisons using the Tukey test (Table 3) showed that FCT and CT had significantly lower internal temperatures than Out. Temp. Notably, CT exhibited the lowest internal temperature, significantly lower than both FCT and Out. Temp. Specifically, CT’s internal temperature was significantly lower than that of FCT, with a mean difference of 1.01 °C. Additionally, FCT’s temperature was lower than Out. Temp. by 1.46 °C, while CT was lower than Out. Temp. by 2.48 °C. Specifically, CT was 1.01 °C lower than FCT, 2.48 °C lower than Outdoor Temp., and FCT was 1.46 °C lower than Outdoor Temp.

Even though the results of the Shapiro–Wilk tests indicated that the data met the normality assumption for the external temperature (W = 0.991, *p* = 0.979), the internal temperature of the MPM Tile (W = 0.956, *p* = 0.089), and the FCT Tile (W = 0.981, *p* = 0.681), the Levene [F(2, 29) = 4.857, *p* = 0.009] test detected variances are unequal.

The one-way ANOVA with Welch correction indicated significant differences in temperature among groups, F(2, 83.376) = 397.19, *p* < 0.001. Post hoc pairwise comparisons using the Games–Howell test presented in Table 4 revealed that MPM had the lowest internal temperature, significantly lower than both Out. Temp. and FCT. Particularly, MPM was 7.92 °C lower than Out. Temp., and 7.17 °C lower than FCT. Moreover, FCT had a significantly lower internal temperature than Out. Temp. by 0.75 °C.

### 3.8. Humidity Impact

The high humidity typical of the Amazon can significantly affect the behavior of natural fibers such as miriti, especially through the absorption of water by the cell wall. This absorption tends to alter the density and internal structure of the fiber, increasing its thermal conductivity and, in some cases, reducing its insulation capacity. In humid tropical climates, the moisture content in the air can reach values above 90%, which favors the penetration of water into the fibers, causing swelling and possible microcracks or voids in the fiber–matrix interface.

In MPM, the polyurethane resin acts as a partial barrier, reducing, but not eliminating, the diffusion of moisture to the fibers. If the blanket is not properly waterproofed or protected, the residual moisture accumulated in the fiber can modify the thermal conductivity and even impact the long-term mechanical durability since cycles of water absorption and release cause dimensional variations. In extreme humidity conditions, the composite may suffer from delamination or loss of localized adhesion, as the swelling of the fibers creates internal stresses that weaken the interface.

Despite this, the choice of miriti fibers, native to the region, brings advantages in adapting to the local climate: the species has developed natural mechanisms of resistance to humidity. PU impregnation also slows down the access of water to the cell walls. In parallel, the use of hydrophobic additives or surface treatments can minimize moisture absorption, helping to maintain low thermal conductivity over time.

### 3.9. Resistance to Ultraviolet Radiation

Polyurethane (PU) obtained from castor oil, although it has advantages as a renewable source and good adhesion to natural fibers, has the weakness of low resistance to ultraviolet (UV) radiation, which can degrade its polymer chain over time. In hot and humid climates like the Amazon, this problem is aggravated by the combination of intense solar radiation, high temperatures, and high relative humidity. To overcome this limitation, two strategies were essentially adopted: firstly, miriti fibers (*Mauritia flexuosa*) were incorporated into the PU matrix, creating a composite with greater dimensional stability and mechanical resistance. The natural fiber acts as a kind of “skeleton” that helps dissipate tension and reduces the resin’s direct exposure to UV rays, in addition to contributing to thermal insulation.

The second strategy involves the application of additives and/or external coatings that act as a protective barrier against solar radiation. The process, a common practice, involves the use of pigments, stabilizers, or varnishes that absorb or reflect UV radiation, inhibiting the photoaging of the PU. Some antioxidant or anti-UV additives can be incorporated directly into the polyurethane formulation before curing, delaying degradation.

Furthermore, in the context of the Amazon, protection against humidity is essential; therefore, the presence of fibers well impregnated with resin and the possible application of hydrophobic external layers help to prevent infiltration and delamination. Together, these solutions—fibrous reinforcement, stabilizing additives, and surface coatings—allow MPM to maintain satisfactory thermal and mechanical performance, even under intense radiation and high humidity, ensuring a longer useful life in the Amazon environment and enabling its use as a thermal roof blanket.

### 3.10. Practical Feasibility

Despite presenting promising thermal performance, MPM needs to meet other safety and durability criteria to be viable in real roofs, especially in tropical climates such as the Amazon. One of the critical factors is fire resistance: as it is an organic material (PU from castor oil and vegetable fibers), it is essential to assess whether it meets the standards for flammability and flame propagation, adopting retardant additives or surface treatments that inhibit rapid burning in the event of a fire. Without these precautions, the risk of ignition and fire spreading may be greater than with ceramic or metal tiles.

The durability of the adhesion between the miriti fiber and the polyurethane is another point that directly influences the useful life of the composite. In environments with high humidity and temperature variations, such as in the Amazon, cycles of water absorption and release can compromise the fiber–matrix interface. To overcome this, surface treatments are often used on the fibers (e.g., alkaline or silane) or additives in the PU itself in order to minimize moisture infiltration and maintain internal cohesion.

As for maintenance, even if the blanket presents good initial thermal performance, it is likely that it will require periodic inspections and possible repairs to ensure the integrity of the composite. Prolonged exposure to UV radiation and acid rain can gradually degrade the PU matrix, reducing thermal efficiency and mechanical strength. Applying protective coatings (varnishes or UV-block paints) and checking for cracks or delamination are recommended procedures to extend the useful life. Thus, although MPM has proven to be effective in thermal insulation, its application on a real scale requires consideration of fire safety standards, durability of fiber–matrix adhesion, and maintenance strategies that ensure physical stability and performance over time.

## 4. Conclusions

In the present work, a composite material was developed from two-component polyurethane (PU) based on castor vegetable oil and reinforced with miriti fiber (*Mauritia flexuosa*) to be used as a thermal blanket. The following conclusions were drawn:

1. Comparative Thermal Performance:
  - In the comparative analysis of internal temperatures, it was observed that fiber cement, ceramic, and metallic tiles with thermal blankets meet the specified standards regarding thermal performance criteria, maintaining the maximum temperatures inside the experimental modules below the temperatures observed for the external environment;
  - The data presented show that there was a lower thermal gain in the module covered with ceramic tiles, representing a thermal efficiency of 2.52% greater than fiber cement tiles. However, the metal tile presented better thermal performance, as it managed to maintain the internal temperature of the module 18.40% below the external (ambient) temperature and 16.66% below the internal temperature of the module covered with fiber cement tiles, showing that due to the application of white paint and thermal blanket, the metal tile was more efficient in its thermal insulation capacity, making it difficult to transmit heat to the interior of the experimental module;
  - It can be stated through the results obtained that, in fact, the fiber cement tile, in addition to being the thinnest and, therefore, the lowest thermal resistant, was the one that transferred the most heat into the experimental module, different from the behavior of the metal tile, which due to the application of the thermal blanket produced from a two-component polyurethane (PU) composite based on castor vegetable oil and reinforced with miriti fiber (*Mauritia flexuosa*), presented better thermal performance than fiber cement and ceramic tiles.
2. Efficiency of the PU/Miriti Composite:
  - The PU/miriti composite has proven to be highly efficient as a thermal insulator due to its multilayer structure and the low thermal conductivity of the vegetable polyurethane. This combination created an effective thermal barrier, reducing the internal temperature range and providing greater thermal comfort;
  - The application of thermal blankets on metal tiles stood out as a sustainable and efficient solution, especially in hot and humid climates, where reducing the thermal load is essential for the comfort of occupants and the energy efficiency of buildings.
3. Advantages and Limitations:
  - Fiber cement, although durable and cost-effective, has proven to be less efficient in extreme weather conditions, requiring complementary strategies, such as thermal blankets or reflective paints, to improve its performance;
  - The PU/miriti thermal blanket, on the other hand, showed superior performance, but its economic viability and long-term durability must be evaluated in comparison with traditional alternatives, considering factors such as acquisition cost, maintenance, and weather resistance.
4. Contributions and Practical Implications:
  - This work contributes to the development of sustainable and energy-efficient construction materials aligned with the demands of tropical climate regions. The combination of a renewable matrix (castor oil) with an abundant natural fiber (miriti) represents a significant advance in the search for environmentally friendly solutions for thermal insulation;
  - The results suggest that the application of PU/miriti thermal blankets on metal tiles can be a viable strategy to reduce energy consumption in refrigeration systems, promoting environmental sustainability and thermal comfort in buildings.
5. Future Perspectives:
  - Additional studies are needed to evaluate the durability of the PU/miriti composite under real-world conditions of use, including prolonged exposure to humidity, ultraviolet radiation, and extreme weather variations;
  - Optimizing the manufacturing process and reducing costs can increase the economic viability of this solution, making it accessible for large-scale applications.

In summary, this study demonstrates that the PU/miriti composite presents superior thermal performance to fiber cement and ceramic tiles, standing out as a promising solution for thermal insulation in hot climate regions. The choice between materials must consider not only thermal performance but also factors such as cost, durability, and specific climatic conditions, aiming to optimize the thermal comfort and environmental sustainability of buildings.

## Figures and Tables

**Table 1:** Thermal performance (reduction, range, and rating) by period and coating.

Period	Coating	Thermal Reduction (°C)	Range (°C)	Performance
August/2022	FCT	3.6634	1.02–1.99	Minimum/Intermediate
	CT	5.9475	2.00–2.99	Superior
September/2022	FCT	3.63291	1.02–1.99	Minimum/Intermediate
	CT	6.13388	2.00–2.99	Superior
October/2022	FCT	3.60598	1.02–1.99	Minimum/Intermediate
	CT	6.37368	2.00–2.99	Superior
November/2023	FCT	1.74124	0.43–1.19	Minimum/Intermediate
	MPM	18.64058	5.18–10.11	Superior
December/2023	FCT	1.74721	0.43–1.19	Minimum/Intermediate
	MPM	17.88021	5.18–10.11	Superior

**Table 2:** Comparison of internal temperatures of this work and others present in the literature.

Material Evaluated	Properties Analyzed	Main Results	Ref.
Fiber cement, ceramic, and metal roof tiles with white paint and two-component polyurethane blanket based on castor oil and miriti fiber	Internal temperature	The PU/miriti blanket had superior thermal performance, reducing the internal temperature by up to 10 °C	This study
Metal tiles with EPS and PU insulation	Heat transfer	PU showed better thermal performance than EPS at high temperatures	[53]
Ceramic, fiber cement, and metal tiles (without roofing)	External and internal temperatures	Metal tiles exceeded 53 °C on the surface, with the worst thermal performance	[54]
Metal and fiber cement roof tiles	Internal temperature	Fiber cement tiles showed better thermal performance compared to metal tiles	[55]
Metal roof tiles without coating, with epoxy coating and with a composite coating of natural andesite stone powder and epoxy	The reduction in internal temperature and the effect of the thickness of the composite coating	The epoxy coating proved effective in reducing the internal temperature	[56]

**Table 3:** Post hoc Tukey results.

Comparison	Mean Difference	95% Conf. Interval		Sig.
		Lower Limit	Upper Limit	
FCT vs. Out. Temp.	−1.463	−1.792	−1.133	<0.001
CT vs. Out. Temp.	−2.476	−2.805	−2.146	<0.001
CT vs. FCT	−1.013	−1.342	−0.684	<0.001

**Table 4:** Post hoc Games–Howell results.

Comparison	Mean Difference	95% Conf. Interval		Sig.
		Lower Limit	Upper Limit	
MPM vs. Out. Temp.	−7.923	−8.613	−7.232	<0.001
MPM vs. FCT	−7.174	−7.884	−6.463	<0.001
FCT vs. Out. Temp.	−0.749	−1.288	−0.210	<0.004

## Data Availability

The original contributions presented in the study are included in the article; further inquiries can be directed to the corresponding author.
